# Supplementary material for: Towards unified AI-driven fracture mechanics: the extended deep energy method (XDEM)
Source: Nat Commun. 2026 Aug 15;17:8492. doi: 10.1038/s41467-026-76748-1 (PMC13484471; doi:10.1038/s41467-026-76748-1)
Supplement: Supplementary file 3 [file 41467_2026_76748_MOESM3_ESM.zip › pic/Results/Stress_intensity_factor/Mixed/K1_K2_single_comparison.pdf]

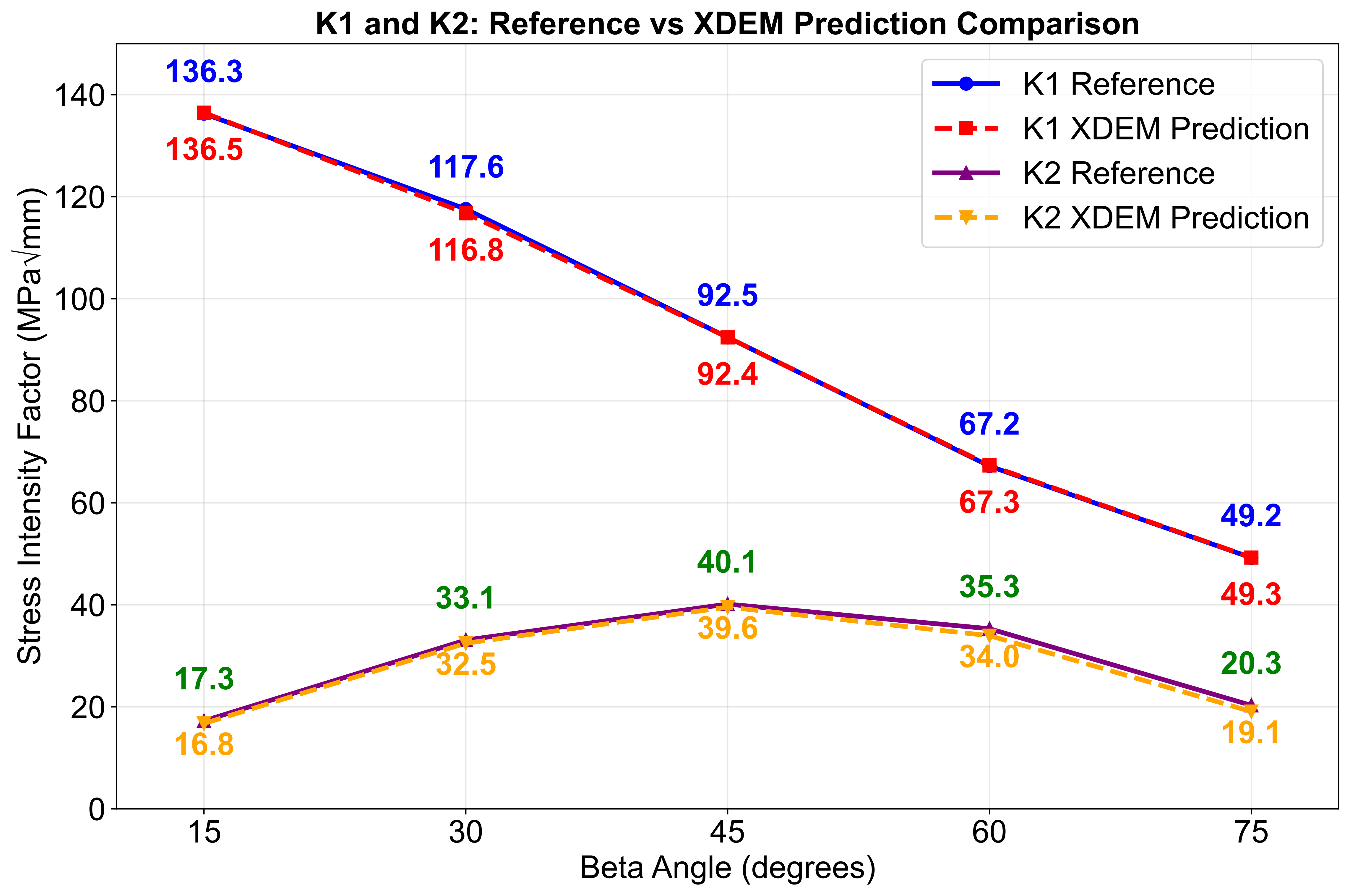
K1 and K2: Reference vs XDEM Prediction Comparison
Stress Intensity Factor (MPa√mm)
Beta Angle (degrees)
0
20
40
60
80
100
120
140
15
30
45
60
75
K1 Reference
K1 XDEM Prediction
K2 Reference
K2 XDEM Prediction
136.3
136.5
117.6
116.8
92.5
92.4
67.2
67.3
49.2
49.3
17.3
16.8
33.1
32.5
40.1
39.6
35.3
34.0
20.3
19.1
